# Revisiting the Role of Carnitine in Heart Disease Through the Lens of the Gut Microbiota

**DOI:** 10.3390/nu16234244

**Published:** 2024-12-09

**Authors:** Jean Demarquoy

**Affiliations:** Unité Mixte de Recherche Procédés Alimentaires et Microbiologiques (UMR PAM), Institut Agro, Institut National de Recherche Pour L’agriculture, L’alimentation et L’environnement (INRAE), Université de Bourgogne, 21000 Dijon, France; jean.demarquoy@u-bourgogne.fr

**Keywords:** carnitine, microbiota, TMAO, heart, health

## Abstract

L-Carnitine, sourced from red meat, dairy, and endogenous synthesis, plays a vital role in fatty acid metabolism and energy production. While beneficial for cardiovascular, muscular, and neural health, its interaction with the gut microbiota and conversion into trimethylamine (TMA) and trimethylamine N-oxide (TMAO) raise concerns about heart health. TMAO, produced through the gut-microbial metabolism of L-carnitine and subsequent liver oxidation, is associated with cardiovascular risks, including atherosclerosis, heart attacks, and stroke. It contributes to cholesterol deposition, vascular dysfunction, and platelet aggregation. Omnivorous diets, rich in L-carnitine, are associated with higher TMAO levels compared to plant-based diets, which are linked to lower cardiovascular disease risks. Dietary interventions, such as increasing fiber, polyphenols, and probiotics, can modulate the gut microbiota to reduce TMAO production. These strategies seek to balance L-carnitine’s benefits with its potential risks related to TMAO production. Future research should focus on personalized approaches to optimize L-carnitine use while mitigating its cardiovascular impacts, exploring microbial modulation and dietary strategies to minimize the TMAO levels and associated risks.

## 1. Introduction

L-Carnitine (β-hydroxy-γ-N-trimethylaminobutyric acid) is a derivative of the amino acid lysine. It was first isolated in 1905 from meat (derived from the Latin term “*carnus*”). Only the L-isomer of carnitine is biologically active. Initially, L-carnitine appeared to function as a vitamin in the mealworm (*Tenebrio molitor*), leading to its designation as vitamin Bt [1]. However, this nomenclature is inaccurate, since humans and other higher organisms are capable of synthesizing L-carnitine endogenously. In certain conditions, the body’s demand for L-carnitine may surpass its synthesis capacity, classifying it as a conditionally essential nutrient [2].

It is synthesized endogenously in the liver and kidneys from the amino acids lysine and methionine [3] and is also obtained from dietary sources, particularly animal products, such as red meat, fish, and dairy products [4]. Carnitine’s primary metabolic role is transporting long-chain fatty acids into the mitochondria for beta-oxidation. The transport of long-chain fatty acids into the mitochondria involves the enzyme carnitine palmitoyltransferase I (CPT I), which catalyzes their transfer to carnitine, forming acyl-carnitine [5]. The acyl-carnitine is then shuttled across the mitochondrial membrane by carnitine-acylcarnitine translocase (CACT). Once inside the mitochondria, carnitine palmitoyltransferase II (CPT II) releases the acyl-CoAs, allowing it to undergo beta-oxidation to produce acetyl-CoA, which enters the citric acid cycle and subsequently generates ATP [6]. The system involving these three proteins is known as the carnitine system.

Beyond its crucial role in fatty acid oxidation, carnitine is also involved in the peroxisomal metabolism of acyl- and acetylCoA and the regulation of the acyl-CoA and CoA ratios, thereby maintaining cellular energy homeostasis [7]. It aids in the excretion of excess acyl groups by forming acyl-carnitines, which are transported out of the mitochondria and excreted in the urine.

In cardiovascular health, carnitine and its derivatives, such as acetyl-L-carnitine, have been shown to improve heart muscle function, reduce ischemia, and protect against oxidative stress [8]. These benefits suggest that carnitine may play a protective role in both managing and preventing cardiovascular diseases. Additionally, carnitine supplementation is associated with enhanced exercise performance and recovery. By facilitating the use of fat as an energy source, carnitine helps to spare muscle glycogen and reduce lactate accumulation, thereby improving endurance and reducing muscle soreness [9].

Acetyl-L-carnitine has been shown to support mitochondrial function and reduce oxidative damage in neural tissue, potentially improving cognitive function in elderly individuals and those with neurodegenerative conditions [10]. It has also been implicated in several individuals with autism, where a defect in L-carnitine has been reported, suggesting that carnitine supplementation may be useful [11,12].

**Figure 1:**
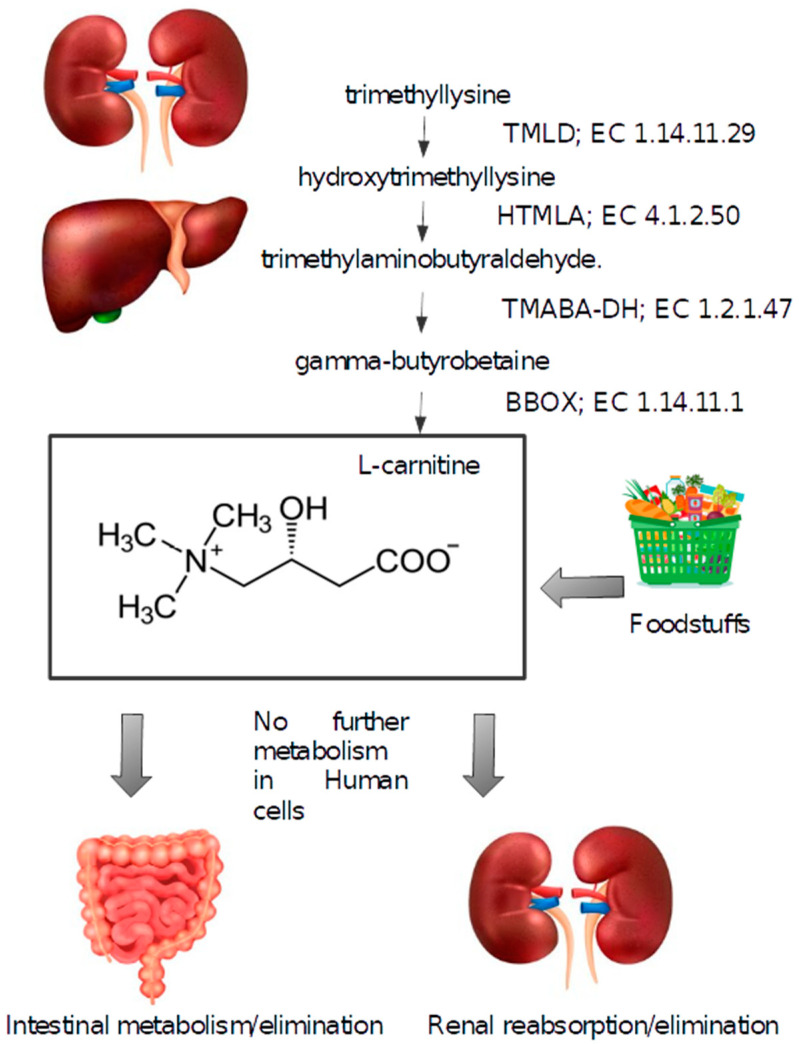
Schematic representation of L-carnitine in the human body. The enzymes involved in its biosynthesis are specified, and their EC numbers are provided. More details regarding the names of the enzymes are presented in the main text. This figure was created using Freepik with images from macrovector and brgfx (https://fr.freepik.com/, accessed on 1 November 2024). TMLD: trimethyllysine dioxygenase; HTMLA: hydroxytrimethyllysine aldolase; TMABA-DH: trimethylaminobutyraldehyde dehydrogenase; BBD: gamma-butyrobetaine dioxygenase.

A recent area of interest for carnitine is the interaction between carnitine and the gut microbiota. Gut microbes metabolize carnitine into trimethylamine (TMA) [13], which is absorbed by enterocytes and converted into trimethylamine-N-oxide (TMAO) in the liver [14]. Elevated TMAO levels have been linked to an increased risk of cardiovascular diseases, indicating a complex relationship between carnitine intake and health outcomes [14,15] (Figure 1).

## 2. Sources and Dietary Intake of Carnitine

L-Carnitine in the human body can come either from endogenous synthesis or dietary sources. For people with a non-vegetarian diet, 75% of the carnitine present in the human body comes from dietary sources [4].

### 2.1. Carnitine Biosynthesis

The synthesis of L-carnitine in the human body primarily occurs in the liver and kidneys, with smaller contributions from the brain and testes. This biosynthesis involves a multi-step process starting from the amino acids lysine and methionine [16].

**Figure 2:**
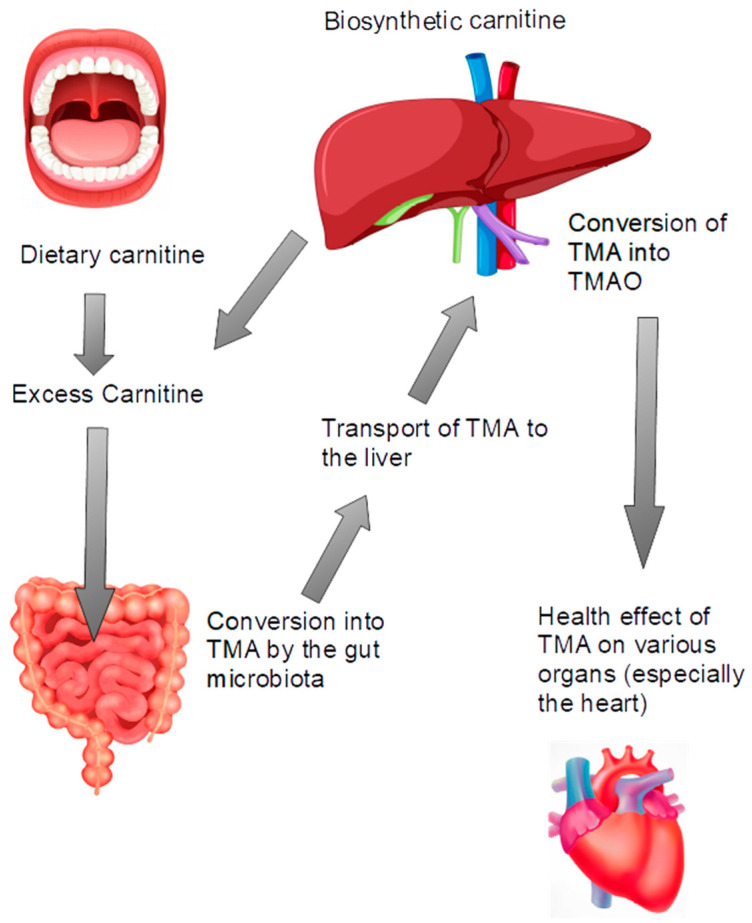
Organs and mechanisms involved in the conversion of carnitine into TMAO. The fate of excess carnitine in the intestine. Carnitine can be converted to TMA by the gut microbiota and transported to the liver, where it is converted to TMAO. TMAO may exert negative effects on various tissue types and organs, especially the heart. This figure was created using Freepik with images from macrovector and brgfx (https://fr.freepik.com/, accessed on 1 November 2024).

Initially, trimethyllysine undergoes hydroxylation by trimethyllysine dioxygenase (TMLD), resulting in the formation of hydroxytrimethyllysine. HTMLA then cleaves this product to form trimethylaminobutyraldehyde, which is oxidized by TMABA-DH to yield gamma-butyrobetaine. Finally, BBH converts gamma-butyrobetaine into L-carnitine [3] (Figure 2).

The synthesis of carnitine can be influenced by the presence of other nutrients, such as vitamin B6, which is essential for carnitine synthesis. Vitamin C is a cofactor for L-carnitine biosynthesis, but its implications appear controversial [17].

### 2.2. Major Dietary Sources of Carnitine

Carnitine is predominantly found in animal-based foods [4,18,19], with veal and red meat being the richest sources (up to 133 mg/100 g). Lamb also has a high carnitine content, up to 40 mg/100 g. Pork also provides a significant amount of carnitine, around 30 to 50 mg per 100 g, varying with the specific cut of the meat. Major data are presented in Table 1.

Dairy products are another significant source of carnitine. For instance, milk contains approximately 2 mg of carnitine per 100 mL, while cheese typically provides smaller amounts than meat but still makes a meaningful contribution to the daily intake. Fish, such as cod and herring, also contain carnitine, although in smaller quantities than red meat. Cod provides around 2 mg per 100 g, and salmon offers about 6 mg per 100 g.

Poultry, including chicken and turkey, contains lower levels of carnitine than red meat. Chicken and turkey typically contain approximately 3 to 10 mg per 100 g [20]. Plant-based sources of carnitine are minimal, except for avocado, containing about 8 mg per 100 g. While plant-based sources contribute only trace amounts, they are still relevant for individuals with plant-based diets.

## 3. Carnitine Metabolism in the Human Body

The bioavailability of carnitine, whether from dietary sources or supplements, plays a crucial role in its effectiveness in the body. Studies have shown varying bioavailability percentages for carnitine intake. For dietary sources, the bioavailability of l-carnitine ranges from 54% to 87% [21]. On the other hand, pharmacological doses of L-carnitine have lower bioavailability, typically between 5% and 18% [21]. The absorption of L-carnitine dietary supplements in doses of 0.5–6 g is primarily passive, with bioavailability of 14–18% of the dose [22]. In individuals adapted to low-carnitine diets, such as vegetarians, the bioavailability of L-carnitine is higher (66% to 86%) compared to regular red meat eaters (54% to 72%) [23].

### 3.1. Absorption and Transport

Carnitine’s metabolism in the human body begins with its absorption in the small intestine. The primary active transport mechanism involves the sodium-dependent transporter OCTN2 (organic cation/carnitine transporter 2), which facilitates the efficient uptake of carnitine into the enterocytes [24]. Once inside the enterocytes, carnitine is transported into the bloodstream and is distributed to the heart, skeletal muscles, and liver, which have high metabolic demands and thus high carnitine requirements.

The renal reabsorption of carnitine is a crucial process in maintaining carnitine homeostasis in the body. The primary mechanism responsible for the reabsorption of carnitine in the kidneys is through the high-affinity carnitine transporter known as organic cation transporter new 2 (OCTN2; SLC22A5) [25]. This transporter is abundantly expressed in the kidneys and plays a significant role in the efficient reabsorption of carnitine from the urine.

The reabsorption of L-carnitine in the kidneys is highly efficient, with 90–99% of the filtered load being reabsorbed under normal circulating levels [26]. This reabsorption process is crucial in conserving carnitine in the body, especially when the dietary intake of carnitine is reduced [26]. OCTN2 is involved in the transport of acylcarnitine, in addition to L-carnitine, contributing to the maintenance of the carnitine levels in the plasma and tissue [27]. In addition, when the circulating L-carnitine concentration increases, as in the case of oral supplementation, the renal reabsorption of L-carnitine may become saturated, resulting in the increased urinary excretion of L-carnitine.

### 3.2. Future of Carnitine in the Human Body

There is no endogenous catabolism of carnitine in human cells. The body’s mechanisms focus on utilizing carnitine for metabolic processes or excreting it when in excess. Carnitine is excreted primarily via the kidneys, with the majority being reabsorbed to maintain homeostasis. Dietary or supplemental L-carnitine that is not absorbed by enterocytes is degraded by colonic bacteria to form two principal products, trimethylamine and γ-butyrobetaine. γ-Butyrobetaine is eliminated in the feces. Any degradation of carnitine into trimethylamine (TMA) occurs through microbial activity in the gut [28]. Trimethylamine is efficiently absorbed and metabolized to trimethylamine-N-oxide, which is excreted in the urine [29].

## 4. From Carnitine to TMAO: The Gut Microbiota

Carnitine’s interaction with the gut microbiota is a complex process that significantly influences human health. When carnitine is ingested, either through the diet or supplementation, part of it reaches the gut, where it can be metabolized by the resident microbiota, which comprises a diverse community of microorganisms, including bacteria, archaea, viruses, and fungi. These play crucial roles in the digestion and metabolism of various nutrients [30], including carnitine.

Carnitine’s metabolism in the gut microbiota involves several key enzymes that convert carnitine into various metabolites, primarily trimethylamine (TMA). Notably, carnitine monooxygenase (CntA) catalyzes the conversion of carnitine into TMA and malic semialdehyde [31], while carnitine dehydratase (CD) facilitates the dehydration of carnitine to produce crotonobetaine [32]; this mechanism has not been extensively studied. Once TMA is produced, it is absorbed into the bloodstream and transported to the liver, where the enzyme flavin-containing monooxygenase (FMO) oxidizes TMA into trimethylamine-N-oxide (TMAO). TMAO is subsequently released into the bloodstream and excreted by the kidneys. Although TMA can also be converted into TMAO by gut-microbial enzymes such as TMA monooxygenase, this pathway is primarily observed in marine bacteria and is considered insignificant in the human gut microbiota [33].

The presence of CntA genes in bacterial genomes highlights the key role of the gut microbiota in TMA production. A study analyzing over 67,000 bacterial genomes identified 6738 genomes with putative CntA genes, predominantly in Proteobacteria, particularly Gammaproteobacteria (e.g., *Escherichia* and *Acinetobacter*). Some entries were also found in Betaproteobacteria and Epsilonproteobacteria [13]. The diet plays a critical role in shaping the microbiota composition and TMAO production. For example, vegan and vegetarian diets are associated with lower plasma TMAO levels compared to omnivorous diets. An analysis of the bacterial 16S rRNA genes in fecal samples from 53 individuals showed a strong association between the microbiota composition and plasma TMAO levels but not with carnitine or choline. *Prevotella*-enriched enterotypes exhibited higher TMAO levels than Bacteroides-enriched ones, demonstrating the influence of dietary habits on the gut microbiota and its capacity to metabolize L-carnitine into TMA and TMAO [34].

The gut microbiota’s composition directly modulates TMA production, with dietary interventions offering potential for mitigation. Methionine restriction has been shown to reduce the TMA levels by altering the gut microbiota composition [35]. Additionally, berberine can attenuate choline-induced atherosclerosis by inhibiting TMA and TMAO production through microbiota manipulation [36]. These findings highlight the interplay between the diet, microbiota, and carnitine metabolism, highlighting the potential for targeted dietary strategies to reduce the TMAO-associated cardiovascular risks.

## 5. Benefits and Risks of Carnitine and TMAO in Cardiovascular Disease

### 5.1. Health Benefits

Clinically, carnitine supplementation has been shown to benefit individuals with specific deficiencies, improving energy metabolism and reducing symptoms of fatigue [9]. Moreover, it is associated with potential cardiovascular benefits, including improved cardiac function and reduced ischemic injury [37,38]. Carnitine supplementation offers numerous health benefits across various conditions. In cardiovascular health, it enhances heart function, reduces symptoms of angina, and improves exercise tolerance, particularly in patients with heart failure and ischemic heart disease [39]. For athletes and physically active individuals, carnitine aids in reducing muscle soreness, minimizing oxidative stress, and enhancing endurance and recovery [40,41]. Additionally, acetyl-L-carnitine has shown promise in supporting cognitive function, especially in aging populations and those with neurodegenerative diseases, by improving mitochondrial function and reducing oxidative damage [11].

For secondary prevention post-myocardial infarction, the daily oral maintenance doses of L-carnitine have ranged from 2 to 6 g. A systematic review and meta-analysis found no significant differences in all-cause mortality among these dosages, suggesting that doses greater than 3 g per day may not provide additional benefits [42]. Most interventional nutritional studies use a carnitine supplementation regimen of 2 to 3 g per day.

The beneficial effects of L-carnitine on cardiovascular health have been extensively studied. Over the past decade, comprehensive meta-analyses have explored its role, highlighting L-carnitine’s multifaceted benefits for cardiovascular health. Shang et al. (2014) showed reduced mortality with high doses of L-carnitine [42]. Similarly, Sahebkar (2015) demonstrated reductions in inflammation markers, such as C-reactive protein, associated with L-carnitine [43]. Song et al. (2017) highlighted its efficacy in chronic heart failure management [39]. A meta-analysis by Abbasnezhad et al. (2020) found that L-carnitine supplementation improved the lipid profiles in patients with liver disease [44]. Additionally, Fathizadeh et al. (2019 and 2020) found, in a series of meta-analyses, reductions in oxidative stress and inflammation, supporting its anti-atherosclerotic potential [45,46,47]. In terms of secondary prevention, Musazadeh et al. (2023) summarized evidence across meta-analyses, confirming its role in lipid management [48]. More recently, Mirrafiei et al. (2024) reported modest improvements in cardiovascular risk factors in adults with type 2 diabetes [49]. Meta-analyses by Asadi et al. (2020) and Anaraki et al. (2023) confirmed L-carnitine’s positive impact on lipid profiles and blood pressure regulation, respectively [50,51]. Collectively, these findings highlight L-carnitine’s therapeutic potential in improving cardiovascular health and mitigating risk factors.

Despite its benefits, high doses of carnitine can cause mild toxicity, with symptoms including muscle weakness and a fishy body odor [52]. Additionally, interactions with certain medications and increased risks for individuals with pre-existing conditions, such as liver or kidney diseases, highlight the need for cautious use and medical consultation [53].

Carnitine supplementation, independently of its conversion to TMAO, has been associated with several adverse effects, particularly when consumed in excess. Studies have shown that carnitine may exacerbate certain metabolic disorders due to its role in fatty acid metabolism, potentially leading to increased oxidative stress and mitochondrial dysfunction under specific conditions [54]. Additionally, Roth et al. (2024) highlighted that high carnitine levels could alter cerebrovascular health by influencing the gut microbiome composition and systemic inflammatory responses, separately from TMAO-mediated pathways. These findings emphasize the need for caution regarding carnitine supplementation, particularly in individuals with preexisting metabolic or cardiovascular vulnerabilities [55].

### 5.2. Cardiovascular Risks 

The cardiovascular risks associated with carnitine consumption are linked to its conversion into TMAO. Trimethylamine N-oxide (TMAO) is a metabolite produced through a well-characterized meta-organismal pathway. Dietary compounds such as choline, phosphatidylcholine, and carnitine, which are highly concentrated in animal-derived foods like meat, liver, and eggs, are metabolized by gut microbes into trimethylamine (TMA). TMA is then converted into TMAO in the host liver by flavin monooxygenases (EC 1.14.13.8) [56,57]. The production of TMAO has been linked to several adverse health effects, particularly concerning cardiovascular health [58]. Elevated TMAO levels are strongly associated with an increased risk of cardiovascular conditions such as atherosclerosis, heart attacks, and strokes, likely due to its role in cholesterol metabolism and vascular dysfunction [59]. TMAO is thought to contribute to cardiovascular disease by promoting cholesterol deposition in the arterial walls, enhancing platelet aggregation, and impairing cholesterol metabolism [60]. This suggests that the microbial metabolism of carnitine to TMA and subsequently to TMAO is a significant pathway linking the diet, gut microbiota, and cardiovascular health [61].

TMAO levels are virtually undetectable in vegetarians and vegans compared to omnivores, and it exerts various negative cardiovascular (CV) effects [62,63,64]. Although the exact host receptor for TMAO has not been identified, studies have shown that TMAO influences cholesterol, sterol, and bile acid metabolism in the liver; suppresses reverse cholesterol transport; induces platelet responsiveness; and causes vascular dysfunction, resulting in a proatherogenic effect (as reviewed by [65]). TMAO also negatively affects ventricular remodeling. Mice treated with TMAO prior to heart failure (HF) development exhibited worsening pulmonary edema, cardiac enlargement, systolic dysfunction, and myocardial fibrosis compared to placebo-treated mice [66,67]. In large human studies, elevated TMAO levels have been independently associated with atherosclerotic heart disease and have been shown to predict major adverse cardiac events [68]. The TMAO levels are also elevated in patients with chronic HF, correlating with the functional class and diastolic dysfunction, but not systolic dysfunction, suggesting that increased venous congestion associated with “backward failure” may significantly alter the gut microbiota (GMB) to promote TMAO production. The positive correlation between TMAO and markers of inflammation and endothelial dysfunction in HF supports the notion that, beyond the traditional “gut hypothesis” of HF, additional mechanisms by which GMB alterations affect cardiac function and prognosis exist. Indeed, the TMAO levels in patients with chronic HF are independently predictive of both short- and long-term mortality [69]. 

Elevated levels of trimethylamine N-oxide (TMAO) have been associated with an increased risk of atherosclerosis, heart attacks, and strokes, raising concerns about the long-term cardiovascular safety of carnitine supplements and L-carnitine-containing foods, such as red meat. However, the primary factor contributing to the adverse health effects of red meat is likely saturated fat, rather than carnitine. While precise quantification remains challenging, evidence suggests that the risks associated with carnitine or its metabolite, TMAO, are significantly lower than those posed by saturated fat in contributing to atherosclerosis and heightened cardiovascular risks in humans. A clear understanding of the relative risk is essential, and the major established coronary risk factors in humans remain well documented [70].

It should be mentioned that carnitine is not the only precursor for TMA and TMAO. TMA precursors also include dietary choline, betaine, and phosphatidylcholine [71].

### 5.3. TMAO and Other Health Conditions

Beyond cardiovascular diseases, elevated TMAO levels have been implicated in other health conditions, particularly chronic kidney disease (CKD) and metabolic disorders. In patients with CKD, the TMAO levels are often significantly elevated due to impaired renal excretion. High TMAO levels in CKD patients have been associated with increased mortality and adverse cardiovascular outcomes, suggesting that TMAO could be a critical factor in the progression of CKD and its complications [72,73]. Moreover, TMAO has been linked to adverse effects on kidney function, potentially exacerbating the decline in renal health [74].

Emerging evidence also suggests a role for TMAO in metabolic disorders such as type 2 diabetes and insulin resistance [75]. Studies have found that elevated TMAO levels correlate with impaired glucose tolerance and insulin signaling. This association suggests that TMAO might contribute to the pathophysiology of diabetes by influencing metabolic pathways and inflammatory processes [76]. Furthermore, research indicates that TMAO could be involved in the regulation of lipid metabolism, contributing to conditions like fatty liver disease and obesity [77].

## 6. Conclusions and Areas for Future Research

Carnitine intake varies naturally from dietary sources, with typical consumption ranging between 20 and 200 mg per day. Supplementation is often used in specific populations and is considered safe at doses of up to 2 g/day. Certain groups may benefit from supplementation, including vegans and vegetarians with lower dietary intake, elderly individuals experiencing reduced endogenous synthesis, and those with medical conditions like genetic carnitine deficiencies or undergoing kidney dialysis [78,79].

When considering TMAO formation, the role of the gut microbiota becomes critical, as elevated TMAO levels are often associated with an increased cardiovascular risk [80]. Recommendations to mitigate these concerns focus on moderating red meat intake, the primary source of carnitine, and encouraging dietary diversity. 

The gut microbiota composition varies widely among individuals, influenced by factors such as one’s diet, age, genetics, environment, medication use, and lifestyle. This variability affects metabolic outputs, including the production of metabolites like trimethylamine (TMA). Specific bacterial taxa, such as those from the genera *Prevotella*, *Clostridia*, and *Lachnospiraceae*, contribute to TMA production; however, their abundance and activity can differ significantly across populations and even within the same individual over time. Additionally, genetic factors, such as polymorphisms in the FMO3 enzyme responsible for converting TMA to TMAO, may further amplify these differences. Combining carnitine-rich foods with polyphenol-rich plants, such as green tea and berries, can inhibit TMA formation. Additionally, fiber-rich diets and probiotics encourage a gut microbiome that is less conducive to TMA production [81].

Several foods and nutrients, especially polyphenols like resveratrol, fiber-rich diets, and some probiotics, have been shown to limit TMAO production.

A few studies have highlighted the efficacy of resveratrol, a natural polyphenol, in mitigating trimethylamine-N-oxide (TMAO) levels. Resveratrol modulates the gut microbiota composition and inhibits the activity of key enzymes, such as CutC and FMO3, involved in TMAO biosynthesis, thereby reducing the circulating TMAO concentrations and associated cardiovascular risks [82]. Synergistic effects have been observed when resveratrol is combined with other polyphenol-rich foods, such as tea extracts, further enhancing its TMAO-lowering potential [83,84]. Clinical trials have validated its role in improving cardiovascular health markers, supporting its potential as a dietary intervention to attenuate the TMAO-associated CVD risks [82,85].

Fiber-rich diets play a crucial role in reducing trimethylamine-N-oxide (TMAO) formation, a metabolite linked to cardiovascular diseases. High dietary fiber intake modulates the gut microbiota composition, suppressing microbial enzymes like TMA lyases, which are responsible for converting dietary choline and carnitine into TMA, the precursor to TMAO. Additionally, fiber promotes the production of short-chain fatty acids (SCFAs), which counteract the inflammation associated with TMAO [86]. 

Some probiotic strains inhibit the microbial production of trimethylamine (TMA), the precursor to TMAO, and promote gut health by enhancing beneficial *lactobacilli* microbial populations. Studies such as [87] have demonstrated that probiotic supplementation reduces the TMAO levels and improves lipid metabolism during atherosclerosis development. These findings highlight the potential of probiotics as a dietary intervention to mitigate the TMAO-associated cardiovascular risks.

In cases where L-carnitine supplementation is needed, the lowest effective dose should be used, especially in individuals with a high CVD risk. Dietary patterns like the Mediterranean diet, rich in fiber and polyphenols, can also support gut health and reduce TMAO formation.

It is also important to distinguish between carnitine’s effects derived from normal dietary intake and those from supplementation, as these differ significantly in terms of their risks and benefits. Dietary carnitine, primarily derived from red meat, is considered safe at typical consumption levels, whereas high doses from supplements may lead to oxidative stress, mitochondrial dysfunction, or systemic inflammation, especially in susceptible populations. Most adverse effects reported in studies arise from supplementation doses far exceeding the dietary levels, highlighting the need for evidence-based guidelines to delineate thresholds for therapeutic versus harmful use. Addressing these aspects would provide a more nuanced analysis, clarifying the context-dependent risks and benefits of carnitine consumption.

Future research should focus on conducting long-term, large-scale studies to better understand the relationship between carnitine supplementation and cardiovascular health. These studies should aim to clarify whether elevated TMAO levels directly contribute to cardiovascular diseases or if they are merely associated with other underlying risk factors. They should also clarify the real role of carnitine in these processes. 

With ongoing advances in research on the gut microbiome and its metabolites, trimethylamine N-oxide (TMAO) has emerged as a significant factor implicated in the progression of heart failure (HF) and is linked to a poor prognosis in HF patients. However, its precise role in the development and progression of HF remains unclear [88]. The precise role of TMAO in heart disease has not yet been clarified. Several hypotheses suggest that TMAO may contribute to heart disease by disrupting the intestinal barrier, inducing chronic inflammation, promoting oxidative stress, impairing mitochondrial function, and increasing hypercoagulability. A recent review detailed all these hypotheses, and the authors concluded that further research is necessary to validate these mechanisms and determine their clinical significance [88]. 

Investigating the specific microbial pathways and genetic factors that influence TMAO production in the gut is crucial. Research should aim to identify which bacterial species are most responsible for TMA production and how dietary and environmental factors modulate their activity. Exploring strategies to modulate the gut microbiota composition to reduce TMA production from carnitine is another important area. This includes the use of probiotics, prebiotics, or even fecal microbiota transplantation [89]. A few years ago, a strategy aimed at limiting TMA production from choline was proposed [90]; a comparable approach could be developed for carnitine-derived TMAO. Additionally, research could investigate the efficacy of compounds that inhibit the microbial enzymes responsible for TMA production. 

This review did not comprehensively address the variability in the individual responses to carnitine supplementation, such as genetic factors or differences in gut microbiota composition, which may significantly influence TMAO production and the associated risks. Additionally, the potential confounding effects of dietary patterns and other lifestyle factors on the observed outcomes were not thoroughly explored. Future studies should address these gaps by incorporating experimental evidence and investigating personalized nutrition strategies to optimize carnitine intake while minimizing the health risks.

## Figures and Tables

**Table 1:** Carnitine content in various foods (data from [4]).

Food Group	Foodstuff	Carnitine mg/100 g (or 100 mL of Liquids)
Meat	Beefsteak	65.0
	Chicken meat	10.4
	Lamb chop	40.5
	Veal sirloin	132.8
	White ham	33.5
Fish, Seafood	Salmon	5.8
	Tuna (cd in water)	1.5
Dairy Products	Milk 4% fat	2.3
	Camembert	14.4
	Mozzarella	6.3
Fruit, Vegetables	Apple	0.2
	Apricot	0.5
	Pear	0.3
	Carrot	0.3
	Lentil	2.1
	Onion	0.7
	Potato (raw)	2.4
